# Reliability of Lower Extremity Muscle Power and Functional Performance in Healthy, Older Women

**DOI:** 10.1155/2021/8817231

**Published:** 2021-02-17

**Authors:** Konstantina Katsoulis, Sunita Mathur, Catherine E. Amara

**Affiliations:** ^1^University of Toronto, Faculty of Kinesiology and Physical Education, 55 Harbord Street, Toronto, Ontario M5S 2W6, Canada; ^2^University of Toronto, Department of Physical Therapy, Toronto, Canada

## Abstract

Evaluation of the long-term reliability of muscle power and functional performance tests in older, healthy adults is warranted since determining whether performance is consistent over longer durations is more relevant for intervention studies. *Objective*. To assess the long-term test–retest reliability of measures of muscle power and lower body functional performance in healthy, nonexercising, older women. *Methods*. Data were derived from a nonexercising control group (*n* = 18; age = 73.3 (3.4) years; height = 159.6 (7.7) cm; body mass = 69.5 (12.7) kg; BMI = 27.3 (4.8)) of a randomized controlled trial of muscle power training in older women. Participants underwent lower extremity muscle power (Biodex) and functional testing (Short Physical Performance Battery, gait speed, 30-second chair stands, stair climbing, and 400-meter walk) at week 0 (baseline), 9, and 15. *Results*. For the upper leg, intraclass correlation coefficients (ICCs) were very high for knee extension power (0.90–0.97) and high to very high for knee flexion power (0.83–0.96). For lower-leg power, ICCs were high to very high for plantar flexion and dorsiflexion (0.83–0.96). ICCs for functional performance were moderate to very high (0.64–0.93). Coefficient of variation of the typical error (CV_TE_) was <10.5% for knee extension/flexion power, 9.9–20.0% for plantar flexion/dorsiflexion power, and 1.9–14.9% for functional performance. Knee extension power and stair climb power demonstrated the highest reliability for muscle power and function, respectively. Mean values did not change over time, with the exception of the chair stands (*p* < 0.05); however, these changes were not considered clinically meaningful. *Conclusions*. The current study provides evidence supporting the long-term reliability of performance assessments of muscle power and lower body functional capacity over a period of up to 15 weeks in healthy, older women.

## 1. Introduction

Functional status is associated with hospitalization, health decline, and death in older adults [1–3]. Since muscle power (the product of force and velocity) is a critical predictor of functioning in older adults [4], the evaluation of physical function and muscle power is especially important in older women who are at a higher risk for age-related functional decline [5]. Reliability data for the more commonly used tests determining functional status in healthy, older adults is lacking. Analysis of video recordings, in other populations, for the assessment of motor skills performance has been shown to be reliable and comparable to live assessment [6] and could also be used to assess the reliability of functional performance in older adults. While studies typically report the short-term (≤ 2 weeks) reliability of functional outcomes [7–9], the long-term reliability (>12 weeks) [10] is needed to consider the potential impact of the typical age-related decline in functional performance in addition to measurement error. Buehring et al. investigated the short and long-term (∼3 months) reliability of functional tests in older (70–95 years), community-dwelling adults [10]. While their data demonstrate stable results in men across all time points for jumping performance, gait speed, 5 chair stands (5 CS), Short Physical Performance Battery (SPPB), and grip strength, the women showed an apparent improvement in 5 CS performance and decreased performance in jump height and grip strength. Thus, while it is not clear whether jump height and grip strength were impacted by age-related declines, there did appear to be sex-related differences in the reliability of functional measures over the course of four months.

Since resistance training is one of the primary recommended treatments for attenuating age-related functional decline [11] and improving muscle power [12], investigating the reliability of measures over durations typically used for training studies (∼3 months [12]) is warranted. Therefore, the purpose of this study was to investigate the long-term (after 9 and 15 weeks) test-retest reliability of muscle power and functional performance tests in healthy, untrained, older women. A secondary purpose was to establish the inter- and intrarater reliability of multiple raters for functional performance using both in-person assessment and video recordings.

## 2. Materials and Methods

### 2.1. Participants

Data for this study come from the nonexercising control group of a randomized controlled trial (functional outcomes and power training in older women: F-POW; NCT02530723). Older women were recruited in the Greater Toronto Area through flyers, e-mail lists, and electronic newsletters. Inclusion criteria consisted of women, ≥ 65 years old, untrained (< 1 hour/week of structured moderate/high intensity exercise and not currently participating in resistance training), and who provided medical clearance from their physician to participate in exercise. Exclusion criteria consisted of osteoporosis, diabetes, uncontrolled hypertension, or a new/unstable condition diagnosed in the last six months. The University of Toronto's Research Ethics Board approved the study (Protocol #27773) which complies with the Declaration of Helsinki. The reliability analysis included 18 women (mean age = 73.3 (3.4) years, mean height = 159.6 (7.7) cm, mean body mass = 69.5 (12.7) kg, and mean BMI = 27.3 (4.8)).

### 2.2. Procedure

The Guidelines for Reporting Reliability and Agreement Studies [13] were followed. Sample size estimation was made using a web-based sample size calculator (https://wnarifin.github.io/ssc/ssicc.html) and methods from Walter et al. [14]. A previous reliability study testing muscle power in older adults [9] reported an intraclass correlation coefficient (ICC) of 0.96. Using the minimum acceptable reliability of 0.75 [15], *α* = 0.05, 80% power, and two repetitions/raters per participant would require a sample size of at least 10 participants.

After obtaining consent, the Geriatric Depression Scale (15 questions), Mini-Mental State Examination, and RAND Physical Functioning Subscale were administered. Using a Jamar dynamometer, seated handgrip strength was measured with the elbow resting on the arm rail and at a 90-degree angle. Participants were instructed to squeeze the dynamometer “as hard as possible” for five seconds. Three attempts with each hand were measured, and the peak value was recorded as hand grip strength (to the nearest kg).

Testing was conducted at 3 time points: 1 (week 0), 2 (week 9), and 3 (week 15). Participants were asked to abstain from physical activity at least 24 hours prior to testing and to be well-hydrated. No other intervention or contact was administered to participants throughout the control period. The participants were asked to maintain their usual physical activity and dietary intake routines for the duration of the study. Only participants with complete functional data for at least 2/3 time points were included in the current analysis.

### 2.3. Body Composition

Body composition was assessed from whole-body scans using a Lunar Prodigy Dual-Energy X-Ray Absorptiometry Scanner (enCORE software, v. 6.50.069, 2002, General Electric Lunar Corporation, Madison, Wisconsin). Fat-free mass (kg) was calculated as the sum of total lean tissue plus bone mineral content. Body fat (%) was calculated as total fat tissue/body mass × 100. Body mass (kg) was obtained using a standard scale.

### 2.4. Muscle Torque and Power

Muscle power was measured using a Biodex isokinetic dynamometer (System 4.0, Shirley, New York) with all testing completed by one rater. A warm-up of usual pace walking within the lab for three to five minutes preceded the testing. The dominant leg was tested, and leg dominance was determined by asking “which leg would you kick a soccer ball with?” Participants were seated upright on the Biodex (hip angle ∼ 90 degrees) with straps across the chest, lap, and testing leg for stability. The dynamometer was aligned with the knee joint axis of rotation, and the cushioned pad of the lever-arm was placed approximately two inches above the lateral malleolus. Participants were instructed to perform all contractions “as hard and as fast as possible,” and motivation was provided throughout the trial. Maximal voluntary isometric contraction (MVIC) of the knee extensors (KE) and knee flexors (KF) were measured at a knee angle of 90 degrees [9]. Each trial consisted of KE MVIC, and a brief rest followed by KF MVIC. Contraction time was five seconds. Three trials were performed with one minute rest between trials. The peak torque for each muscle group was recorded as MVIC (Newton-metres, Nm). After MVIC testing, 40% of MVIC was calculated and used as the load intensity for testing muscle power using the isotonic mode of the Biodex. Ten isotonic contractions were performed combining both concentric KE and KF back-to-back (10 reps for each) using the participant's full range of motion. Two trials were completed (2 × 10 repetitions) and separated by a two-minute rest. Subsequently, the same procedure was followed to test MVIC and power for plantar flexion (PF) and dorsiflexion (DF).

For PF and DF testing, the Biodex seat was reclined (hip angle ∼110 degrees). The right ankle was tested in all participants. The right leg was elevated such that the tibia was parallel to the floor (knee angle ∼ 145 degrees). The right foot was strapped onto the foot plate, and the dynamometer axis of rotation was aligned with the lateral malleolus and support placed under the hamstrings. MVIC was tested in the neutral position (0 degrees), and PF and DF power were tested within a 30-degree range of motion (from 30 degrees of PF to 0 degrees). The torque (Nm) and instantaneous velocity (rad/s), recorded using external software (Acqknowledge 4.1.1: Biopac Systems Inc., 2010), were multiplied to yield power (W) for each repetition. The highest of the 20 repetitions for each muscle group was used for analyses.

### 2.5. Functional Performance

Functional performance was tested using 6 tests: SPPB, 4-meter fast-pace walk (4 MFP), the 30-second chair stands (30 sCS), stair climbing at usual (SCUP) and fast (SCFP) pace, and 400-meter walk (400 MW). A Sportline Tough Timer Stopwatch (E & B Exercise, LLC, Yonkers, NY, USA) was used by raters for timing. The tests were video-recorded on an iPad mini for all three time points. Functional raters (*n* = 3) were experienced graduate students, trained by the PI. All functional raters were blinded to group allocation and the health history of the participants for the duration of the study. Functional performance was tested by a single rater at each testing session.

### 2.6. Inter- and Intrarater Reliability

The videos from the functional testing at time point 1 were used to assess inter- and intrarater reliability. To assess the interrater reliability, video recordings were viewed and scored to produce video-recorded scores by all three raters. The raters were unaware of the scores recorded by the other raters.

To assess intrarater reliability, the scores from the in-person (live) functional testing at time point 1 were compared with the video-recorded scores of the same rater. Intrarater reliability was compared for 2 raters only (rater 3 was omitted since this rater only tested 2 participants at time point 1). Rater 1 did not complete the 400 MW video analyses. Therefore, intrarater 400 MW data came from rater 2 only. The same stopwatch was used to score functional performance while viewing the video recordings.

### 2.7. Short Physical Performance Battery

The SPPB [16] consists of three components: (1) balance (feet together, semitandem, and tandem); (2) timed 4-meter walk at usual pace (4 MUP) starting with the toes at the start line and finishing once the first foot has completely crossed the 4-meter line; (3) time to complete 5 CS as fast as possible, starting from sitting with feet flat on the floor and arms crossed on the shoulders. Time was started upon rising from the chair and ended after the 5th chair stand. A standard firm chair without handrails with a height of 45 inches was used for all chair testings. The faster of two trials (separated by ∼30-second rest) for the 4 MUP and 5 CS were rounded to the nearest 0.1 seconds and were used for analysis. The score (0–4) for the three components was summed for the SPPB total score (0–12).

### 2.8. Four-Meter Fast Pace Walk

For the 4 MFP [17], participants were asked to stand approximately three meters behind the start line and walk at a maximal pace until they were approximately three meters passed the finish line. Timing (to the nearest 0.1 seconds) started when the foot crossed the starting line and stopped when the foot crossed the finish line.

### 2.9. Thirty-Second Chair Stands

Starting from sitting with feet flat on the floor and arms crossed at the wrists on the shoulders, participants were instructed to perform as many full chair stands as possible in 30 seconds [18]. Only one trial was performed. Timing started upon rising from the chair and chair stand count ended at 30 seconds. If the participant was at least halfway to completing the last chair stand at the 30-second mark, that chair stand was counted in the total number recorded.

### 2.10. Stair Climbing

A 13-step staircase with handrails in a well-lit area was used for stair ascent. Step height averaged 18 centimeters and step width averaged 28 centimeters. The total vertical height of the steps was 2.34 meters. Participants were asked to stand at the base of the stairs with feet together and to grab the handrail if necessary during ascent. Upon the instruction “ready, set, go,” timing (to the nearest 0.1 seconds) started when the first foot left the ground and stopped when both feet were placed at the top of the 13th step. Stair ascent was measured during a “usual” pace (SCUP) and during a “fast” pace (SCFP) with instructions to ascend “as quickly and as safely as possible.” The faster of two trials for each test was chosen for analysis. Stair climb power (SCP) for SCFP was calculated as SCP (W) = ((body mass in kg) × (9.8 m/s^2^) × (stair height in meter))/(time in seconds) [19].

### 2.11. Four-Hundred-Meter Walk

The 400 MW test [20] consisted of 10 laps around cones placed 20 meters apart in a corridor. Participants were instructed to walk “as quickly and as safely as possible.” After a two-minute walking warm-up, time to complete 10 laps was recorded (to the nearest 0.1 seconds). Timing began when the participant lifted their foot off the ground at the starting line and ended when the first foot crossed the line at the end of the 10th lap. Standard encouragement was provided.

### 2.12. Statistical Analysis

Means and SD were calculated. Normality of data was tested with the Shapiro-Wilk test [21] and was visually inspected. The repeated measures analysis of variance (for normally distributed data) and Friedman test (for nonnormally distributed data) were used to test for differences in the means across time points. Intraclass correlation coefficients (ICC) using two-way mixed effects with absolute agreement were calculated for inter/intrarater reliability and time points 1 and 2; 2 and 3; 1 and 3. Thresholds to describe ICCs were very low = < 0.20, low = 0.20–0.50, moderate = 0.50–0.75, high = 0.75–0.90, very high = 0.90–0.99, and extremely high = > 0.99 [15]. Statistical analyses were performed using IBM SPSS (v. 24, New York, USA). Coefficient of variation of the typical error (CV_TE_) [22] was calculated with Microsoft Excel (v. 15.0.5233.1000, Washington, USA) using the standard deviation of the change score/average means of both time points × 100 for time points 1 and 2, 2 and 3, and 1 and 3. Effect sizes were calculated using means and SD of change scores for post-hoc matched pairs statistical tests using G∗Power Statistical Software [23] for time points 1 and 2, and 1 and 3. Bland-Altman plots were created using SigmaPlot (version 14, Illinois, USA) with time points 1 and 3 only (for brevity). Simple linear regression was performed using SigmaPlot with the difference score (time points 3–1) as the dependent variable and the mean score (time point (1 + 3)/2) as the independent variable for all measures to test for bias in these variables.

## 3. Results

Twenty-one participants were randomized to the control group. Three participants discontinued participation after baseline testing (two did not like the group allocation and one indicated a lack of time). Thus, a total of 18 participants were included in the reliability study. For the 18 participants included, two participants dropped out after the midpoint testing (reasons included a lack of time and change in health status) and one participant completed the third functional testing session but did not return for the dynamometer testing (due to travel). Dynamometer data are missing for the 2nd time point for one participant (due to travel), power data are missing for one participant at time point 3 (technical issues), and stair climb data are missing for one participant at time point 1 (technical issues). Thus, the number of participants that completed all three testing time points was 16/18 for functional testing and 13/18 for muscle power. Baseline participant characteristics are listed in Table 1. Overall, the participants were high-functioning (median RAND physical functioning score = 85 and median Mini-Mental State Examination score = 30) and at low risk for depression (median Geriatric Depression Scale score = 0). Mean blood pressure was normal (120/78 mm Hg), and most participants were taking only one medication. Means (SD) for all participants that were included (*n* = 18), regardless of missing data, are available online in Tables S1 (torque and power) and S2 (functional performance). The complete data (means and SD) that were used in statistical testing are listed in Table 2 (torque, *n* = 14; power, *n* = 13; physical function, *n* = 16).

### 3.1. Inter- and Intrarater Reliability

Interrater reliability data are reported in Table 3 and indicated very high to extremely high [15] reliability with ICCs ranging from 0.95 to 1.00 (*p* < 0.05). Intrarater reliability (Table 4) demonstrated high to very high ICCs ranging from 0.85 to 1.00 (*p* < 0.05).

### 3.2. Torque and Power

There were no significant differences in means for torque and power across time points (*p* ≥ 0.05; Table 2). For torque and power of the knee extensors and flexors, CV_TE_ between all time points ranged from 5.7% to 10.5%. CV_TE_ for the plantar flexors and dorsiflexors ranged from 9.7 to 20.0% (Table 5). ICCs for all measures of torque and power (*p* < 0.01) were ≥0.90 indicating very high reliability, with the exception of dorsiflexion power from time points 2 and 3 and 1 and 3 (ICCs = 0.83 and 0.88, respectively; Table 5) where reliability was considered high. Bland-Altman plots can been found in Figures S1–S5 online. The majority of the data fell within the limits of agreement. In addition, there were no significant regression models (all *p* ≥ 0.05, data not shown), suggesting no bias in the data.

### 3.3. Functional Performance

For balance, almost all participants received maximal scores (4/4) at all time points (data not shown). There were no significant differences in the means of the functional performance tests across time points (*p* ≥ 0.05; Table 2) except for the chair stands (5 CS and 30 sCS) which improved significantly from time point 1 to 3 (*p* < 0.05). CV_TE_ for functional performance ranged from 1.9 to 14.9%, and ICCs (*p* < 0.01) ranged from 0.64 to 0.95 reflecting moderate to very high reliability (Table 6). Stair climb power demonstrated the highest reliability, with ICCs of 0.93–0.95 for all time point comparisons. Bland-Altman plots are shown in Figures S6–S14. Regression models for the 30 sCS [*R*^2^ = 0.29, *F*(1,15) = 5.80, *p*=0.03] and the 400 MW [*R*^2^ = 0.29, *F* (1,15) = 5.82, *p*=0.03] were significant.

## 4. Discussion

The main findings include the following: (1) isotonic muscle power demonstrated high to very high reliability over a nine- and fifteen-week duration with knee extension power being the most stable measure; (2) all of the functional tests studied demonstrated reliability that ranged from moderate to high, with stair climb power exhibiting the highest reliability over durations that are commonly used in training studies for older adults [12]; (3) standardized testing protocols, despite having multiple raters and minimal participant familiarization, yielded consistent performance measurements.

The current study utilized the isotonic mode of the Biodex dynamometer to evaluate muscle power and hence used a less common velocity-dependent power measure where movement velocity is measured and the load used can be quantified [25]. The current results for KE power are similar with a previous investigation in older men and women investigating the one-week test-retest reliability of KE power using the isotonic mode [9]. The ICC reported in that study using a similar intensity (50% of MVC) of muscle power was 0.96 (CI = 0.94–0.98), comparing well with the current study's ICCs (0.96–0.97 for all time point comparisons). Thus, KE power appears to be well maintained in untrained, healthy older women over a duration of 15 weeks. For PF and DF power, the ICCs ranged from 0.83 to 0.96, indicating high to very high reliability. These findings are in agreement with Webber et al. who investigated the reliability of lower-leg muscle performance testing in older women using the isotonic mode of a dynamometer over a one-week test-retest interval [22]. They tested PF and DF power at 50% of MVC, and reported ICCs of 0.92 (CI = 0.68–0.93) for PF power and 0.95 (CI = 0.90–0.98) for DF power, with a CV_TE_ of 14 and 12%, respectively. The current study did not determine one-week test-retest results; however, in the shorter test-retest period (nine weeks), PF power ICC was 0.96 (CI = 0.89–0.99, CV_TE_ = 9.9%), and DF power ICC was 0.92 (CI = 0.77–0.97, CV_TE_ = 15.2%). Therefore, the very high test-retest reliability of lower-leg isotonic power previously demonstrated over one week is maintained over nine weeks in a healthy older population.

The current study's ICCs for functional performance testing ranged from 0.64 to 0.95, indicating moderate to very high reliability, and all CV_TE_ were <15% over 15 weeks. In a 2013 systematic review of the properties of physical performance measures in community-dwelling older adults, the SPPB was recommended for use since it was found to have good intrarater reliability (ICCs > 0.7 for total score, 4 MUP, and 5 CS) and validity [26]. Mijnarends et al. reported several short-term reliability statistics for several functional tests, including but not limited to walking tests varying in length from two meters to one kilometer (ICC = 0.94), the 6-minute walk test (ICCs = 0.88–0.94), and the 30 sCS (0.84–0.92), which all reflect high to very high consistency. The current study also demonstrates high to very high reliability in most functional performance tests, with some tests (e.g., walking tests) demonstrating moderate reliability over 15 weeks (Table 6). Although these ICCs were moderate, the low CV_TE_ (<9.6%, Table 6) and consistency in the means (*p* > 0.05, Table 2) support their use for long-term reliability. Only one study from the review by Mijnarends et al. investigated the long-term reliability of the SPPB [27] (participants were moderately to severely disabled women) and reported average ICCs for SPPB score that declined gradually over time from six months (ICC = 0.77) to 36 months (ICC = 0.51). Their six-month average ICC for SPPB score is slightly lower compared with the current study's average ICC (0.87, range = 0.82–0.90). Thus, while a number of studies have reported strong short (∼1–2 week) test-retest reliability of functional performance in older clinical populations with ICCs ranging from 0.76 to 0.95 [19, 27, 28], the reliability of functional performance measures appears to be compromised over longer durations in older adults with physical limitations. While it might be expected that healthy older adults will exhibit more stable functional performance over time compared with a clinical population, the well-documented age-related declines in muscle mass [29], strength, and power [30] call into question over what period of time functional performance remains stable in this population. The present study provides evidence that a variety of functional tests which are commonly used in older adults can remain relatively stable over ∼4 months. However, studies investigating the reliability of these tests over longer durations (≥ 6 months) are needed.

The means for power and functional performance across time points did not change significantly, with the exception of the chair stand tests. There were small but significant changes that occurred from time points 1 and 3 for the 5 CS (faster time: −1.4 seconds) and the 30 sCS (increase in chair stands: 1.6 reps). These average changes are less than the estimated minimally important changes found in the literature for clinical populations, which were 1.7 seconds for the 5 CS [31] and 3.3 chair stands for the 30 sCS [32]. This suggests that although a learning effect might have occurred which resulted in improved chair stand performance, these changes were not clinically meaningful. In agreement with these findings, another investigation found that chair stand performance improved significantly in older women over ∼4 months; however, it remained stable in older men [10]. Chair stand tests might represent a more ambiguous performance test where more practice is needed initially to decrease small “practice effects,” at least in older women. Data from the Lifestyle Interventions and Independence for Elders Pilot Study [33] reported meaningful changes of 0.4–1.5 units for the SPPB, 0.08 meters/second for the 4 MUP, and 50–60 seconds for the 400 MW. Using those estimates, the mean changes in the current study's measures from time points 1 and 3 (0 units, 0.05 meters/second, and 11 seconds for the SPPB, 4 MUP, and 400 MW, respectively) would not be considered meaningful (*p* > 0.05 for mean changes, Table 2). Although the linear regression models for the 30 sCS and 400 MW were significant, suggesting proportional bias, the variance explained for both models was small (29%), and the regression tests were underpowered, limiting interpretation. Taken together, the functional performance data presented in the current study demonstrated trivial changes, not considered clinically meaningful, supporting their consistency as a whole in older women over 15 weeks.

The inter/intrarater analyses produced ICCs that were high to extremely high, supporting the use of video-recorded functional performance assessment in older adults. Our findings are in agreement with other studies in clinical populations that have investigated the reliability of assessing functional performance remotely using video technology versus face-to-face testing [34, 35]. For example, Cabana et al. reported very high to excellent ICCs for interrater (0.95–0.99) and intrarater (0.96–0.99) reliability for the 6-minute walk test and the Timed Up and Go test in a group of stable heart failure patients that were tested in-person as well as remotely through video recordings. Aside from a few technical issues in recording functional performance (e.g., the recording stopped before the termination of a test), and ensuring sufficient lighting, test-marker placement, recording-device placement, and video-storage space, capturing functional performance in videos with the tests used in the current study provided a valid method to measure function that was reproducible (independent of the rater) and highly comparable to live testing. Thus, if standard procedures are followed, it is possible that videos captured by practitioners or participants themselves could be analysed to evaluate and monitor functional performance in older adults, thereby reducing the need for in-person testing.

## 5. Limitations

The current study's participants represent high-functioning, community-dwelling older women (baseline SPPB median score = 12 units), and interpretation of results might not apply to clinical populations or frail older women, which is a limitation. In addition, the change in the mean for the chair stand tests (5 CS and 30 sCS) does impact the interpretation of the stability of these tests over 15 weeks in healthy, older women. The increase in chair stand performance, while not clinically meaningful, does reduce the long-term reliability of this measure and suggests a potential learning effect.

## 6. Conclusions

Lower extremity muscle power and functional tests were found to be reliable in older, healthy women over 15 weeks, given a standardized testing environment and administration, minimal familiarization in study design, and multiple raters. These measures were stable over time and can be used to detect changes in response to interventions in older, healthy women. Future research should examine the long-term reliability of functional test performance in healthy, older men, as well as clinical populations.

## Figures and Tables

**Table 1:** Baseline characteristics of participants (*n* = 18).

	Mean (SD)	Median	(Minimum, maximum)
Age (y)	73.3 (3.4)	74.3	(66.9, 79.5)
Height (cm)	159.6 (7.7)	161.0	(142, 170)
Body mass (kg)	69.5 (12.7)	69.0	(48.4, 96.2)
BMI (kg/m^2^)	27.3 (4.8)	26.6	(19.9, 38.2)
Body fat (%)	40.0 (8.2)	41.0	(28.0, 56.5)
Fat-free mass (kg)	39.8 (7.4)	40.5	(18.7, 52.4)
Hand grip strength (kg)	26.5 (5.4)	27.0	(16.0, 36.0)
Systolic BP (mm Hg)	119.5 (10.4)	120.0	(95, 140)
Diastolic BP (mm Hg)	77.9 (7.7)	77.5	(65, 90)
RAND Physical Functioning Subscale		85	(40, 100)
Geriatric Depression Scale		0	(0, 5)
Mini-Mental State Examination		30	(29, 30)
Medications (#)		1	(0, 10)

^a^Data are means (SD), except for the RAND Physical Functioning Subscale, Geriatric Depression Scale, Mini-Mental State Examination, and medications, where median (min, max) are reported since data were not normally distributed; BMI = body mass index, PF = physical functioning, BP = blood pressure.

**Table 2:** Muscle torque (*n* = 14), power (*n* = 13), and functional performance (*n* = 16) for time points 1 (week 0), 2 (week 9), and 3 (week 15).

	Time point			*p* value	ES 1 and 2	ES 1 and 3
	1	2	3			
KET	92 (35)	92 (26)	92 (28)	0.98	0.05	0.02
KEP	158 (43)	162 (39)	164 (38)	0.29	0.37	0.38
KFP	125 (31)	128 (36)	132 (32)	0.37	0.20	0.33
PFP	109 (36)	104 (37)	112 (40)	0.35	0.32	0.16
DFP	12 (4)	12 (5)	11 (3)	0.47	0.12	0.26
SPPB	12 (9, 12)	12 (9, 12)	12 (9, 12)	0.06	0.20	0.41
4 MUP (s)	3.4 (0.5)	3.2 (0.5)	3.3 (0.5)	0.40	0.27	0.30
4 MFP (s)	2.1 (0.3)	2.2 (0.3)	2.1 (0.3)	0.55	0.15	0.09
400 MW (s)	279.2 (26.5)	271.8 (15.3)	268.3 (16.0)	0.09	0.37	0.50
5 CS (s)	10.5 (2.8)	9.7 (1.6)	9.1 (2.2)^∗^	0.02	0.36	0.76
30 sCS (reps)	14 (3)	15 (3)	16 (5)^∗^	0.01	0.18	0.72
SCUP (*s*) (*n* = 15)	7.9 (1.2)	7.7 (1.3)	7.6 (1.3)	0.44	0.19	0.35
SCFP (*s*) (*n* = 15)	6.0 (1.1)	6.0 (0.9)	6.0 (1.0)	0.98	0.03	0.05
SCP (W) (*n* = 14)	273.3 (76.7)	263.2 (57.0)	271.2 (67.9)	0.51	0.26	0.06

^a^Data are mean (SD), except for the SPPB, where medians (min, max) are displayed since data were not normally distributed; repeated measures ANOVA used to test means across time points, except for the SPPB which was tested using Friedman test; ES = Cohen's *d* effect size for time points 1 and 2 and 1 and 3, except for the SPPB which was calculated using *z*/√*N* for nonparametric data [24]. KET: isometric knee extension torque; KEP: knee extension power; KFP: knee flexion power; PFP: plantar flexion power; DFP: dorsiflexion power; SPPB: Short Physical Performance Battery; 4 MUP: 4-meter usual pace walk; 4 MFP: 4-meter fast pace walk; 400 MW: 400-meter walk; 5 CS: 5 chair stands; 30 sCS: 30-second chair stands; SCUP: stair climb usual pace; SCFP: stair climb fast pace; SCP: stair climb power for the SCFP; ^*∗*^*p* < 0.05. For the 5 CS and 30 sCS, time point 3 was significantly different from time point 1.

**Table 3:** Interrater reliability of functional performance testing for three raters for video-recorded scores for time point 1 (*n* = 17).

Test	ICC	95% CI	*p* value
4 MUP	0.95	0.87–0.98	<0.001
4 MFP	0.98	0.94–0.99	<0.001
5 CS	0.97	0.72–0.99	<0.001
30 sCS	1.00	0.99–1.00	<0.001
SCUP	0.97	0.92–0.99	<0.001
SCFP	0.99	0.97–1.00	<0.001
400 MW (*n* = 15)	1.00	1.00–1.00	<0.001

^a^ICC: intraclass correlation coefficient; CI: 95% confidence intervals; 4 MUP: 4-meter usual pace walk; 4 MFP: 4-meter fast pace walk; 5 CS: 5 chair stands; 30 sCS: 30-second chair stands; SCUP: stair climb usual pace; SCFP: stair climb fast pace; 400 MW: 400-meter walk. All ICCs were significant; ^*∗*^*p* < 0.05.

**Table 4:** Intrarater reliability of functional performance testing between video-recorded scores and live data collection for two raters for time point 1; rater 1 (R1): *n* = 9 participants; rater 2 (R2): *n* = 6 participants.

Test	Rater	ICC	95% CI	*p* value
4 MUP	R1	0.85	−0.13–0.97	<0.001
	R2	0.85	−0.01–0.98	0.01

4 MFP	R1	0.92	0.65–0.98	<0.01
	R2	0.89	0.24–0.99	0.02

5 CS	R1	0.99	0.80–1.00	<0.001
	R2	1.00	0.97–1.00	<0.001

30 sCS	R1	0.99	0.95–1.00	<0.001
	R2	1.00	0.98–1.00	<0.001

SCUP	R1	0.94	0.68–0.99	<0.001
	R2	0.95	0.69–0.99	<0.01

SCFP	R1	0.98	0.93–1.00	<0.001
	R2	0.98	0.50–1.00	<0.001

400 MW (*n* = 4)	R2	1.00	1.00–1.00	<0.001

^a^ICC: intraclass correlation coefficient; CI: 95% confidence intervals; 4 MUP: 4-meter usual pace walk; 4 MFP: 4-meter fast pace walk; 5 CS: 5 chair stands; 30 sCS: 30-second chair stands; SCUP: stair climb usual pace; SCFP: stair climb fast pace; 400 MW: 400-meter walk. All ICCs were significant; ^*∗*^*p* < 0.05.

**Table 5:** Reliability data for muscle torque (Nm) and muscle power (W) for time points 1 and 2 (weeks 0–9; *n* = 17), 2 and 3 (weeks 9–15; *n* = 13), and 1 and 3 (weeks 0–15; *n* = 14).

	CV_TE_			ICC (CI) *p* value		
	1 and 2	2 and 3	1 and 3	1 and 2	2 and 3	1 and 3
KET (Nm)	9.5	8.0 (*n* = 14)	10.1 (*n* = 15)	0.96 (0.89–0.99) <0.001	0.97 (0.89–0.99) <0.001	0.96 (0.87–0.99) <0.001
KEP (W)	5.7	6.1	6.6	0.97 (0.92–0.99) <0.001	0.97 (0.90–0.99) <0.001	0.96 (0.88–0.99) <0.001
KFP (W)	9.7	6.7	10.5	0.93 (0.80–0.97) <0.001	0.97 (0.90–0.99) <0.001	0.90 (0.68–0.97) <0.001
PFP (W)	9.9	14.0	15.2	0.96 (0.89–0.99) <0.001	0.92 (0.74–0.98) <0.001	0.91 (0.72–0.97) <0.001
DFP (W)	15.9	20.0	14.8	0.92 (0.77–0.97) <0.001	0.83 (0.47–0.95) 0.002	0.88 (0.62–0.96) <0.001

^a^CV_TE_: coefficient of variation of the typical error (%); ICC: intraclass correlation coefficient; CI: 95% confidence interval; KET: isometric knee extension torque; KEP: knee extension power; KFP: knee flexion power; PFP: plantar flexion power; DFP: dorsiflexion power. All ICCs were significant; ^*∗*^*p* < 0.05.

**Table 6:** Reliability data for functional performance for time points 1 and 2 (weeks 0–9, *n* = 18), 2 and 3 (weeks 9–15, *n* = 16), and 1 and 3 (weeks 0–15, *n* = 16).

	CV_TE_			ICC (CI) *p* value		
	1 and 2	2 and 3	1 and 3	1 and 2	2 and 3	1 and 3
SPPB	3.6	3.5	3.8	0.90 (0.73–0.96) <0.001	0.88 (0.65–0.96) <0.001	0.82 (0.44–0.94) <0.001
4 MUP (s)	10.1	8.7	9.6	0.76 (0.36–0.91) <0.01	0.81 (0.45–0.93) <0.01	0.74 (0.29–0.91) <0.01
4 MFP (s)	10.3	5.7	9.1	0.77 (0.40–0.91) <0.01	0.91 (0.73–0.97) <0.001	0.69 (0.10–0.89) 0.02
400 MW (s)	5.0	1.9	5.6	0.87 (0.66–0.95) <0.001	0.93 (0.80–0.98) <0.001	0.64 (0.05–0.87) 0.02
5 CS (s)	14.9	10.3	12.7	0.71 (0.25–0.89) 0.01	0.84 (0.57–0.95) <0.001	0.81 (0.30–0.94) <0.001
30 sCS (reps)	9.2	11.8	10.6	0.90 (0.75–0.96) <0.001	0.85 (0.57–0.95) <0.001	0.88 (0.52–0.96) <0.001
SCUP (s)	9.6 (*n* = 17)	6.8	7.8 (*n* = 15)	0.88 (0.65–0.96) <0.001	0.91 (0.73–0.97) <0.001	0.86 (0.60–0.95) <0.001
SCFP (s)	7.9 (*n* = 17)	8.6	9.5 (*n* = 15)	0.93 (0.82–0.98) <0.001	0.80 (0.42–0.93) <0.01	0.82 (0.45–0.94) <0.01
SCP (W)	9.8 (*n* = 16)	6.9 (*n* = 15)	8.9 (*n* = 15)	0.93 (0.80–0.98) <0.001	0.95 (0.86–0.98) <0.001	0.94 (0.82–0.98) <0.001

^a^CV_TE:_ coefficient of variation of the typical error (%); ICC: intraclass correlation coefficient; CI: 95% confidence interval; SPPB: Short Physical Performance Battery; 4 MUP: 4-meter usual pace walk; 4MFP: 4-meter fast pace walk; 400 MW: 400-meter walk; 5 CS: 5 chair stands; 30 sCS: 30-second chair stands; SCUP: stair climb usual pace; SCFP: stair climb fast pace; SCP: stair climb power for the SCFP; all ICCs were significant; ^*∗*^*p* < 0.05.

## Data Availability

The data used in the study are available from the corresponding author upon request via email.

## Supplementary Materials

Supplementary Materials The means (SD) for all participants (regardless of missing data) are shown in Tables S1 (torque and power) and S2 (functional performance). Bland-Altman plots for all measures using time points 1 and 3 are shown in Figures S1–S14.
