# Supplementary material for: Reliability of Lower Extremity Muscle Power and Functional Performance in Healthy, Older Women
Source: J Aging Res. 2021 Feb 17;2021:8817231. doi: 10.1155/2021/8817231 (PMC7904351; doi:10.1155/2021/8817231)
Supplement: Supplementary Materials — The means (SD) for all participants (regardless of missing data) are shown in Tables S1 (torque and power) and S2 (functional performance). Bland-Altman plots for all measures using time points 1 and 3 are shown in Figures S1–S14. [file 8817231.f1.pptx]

## Slide 1
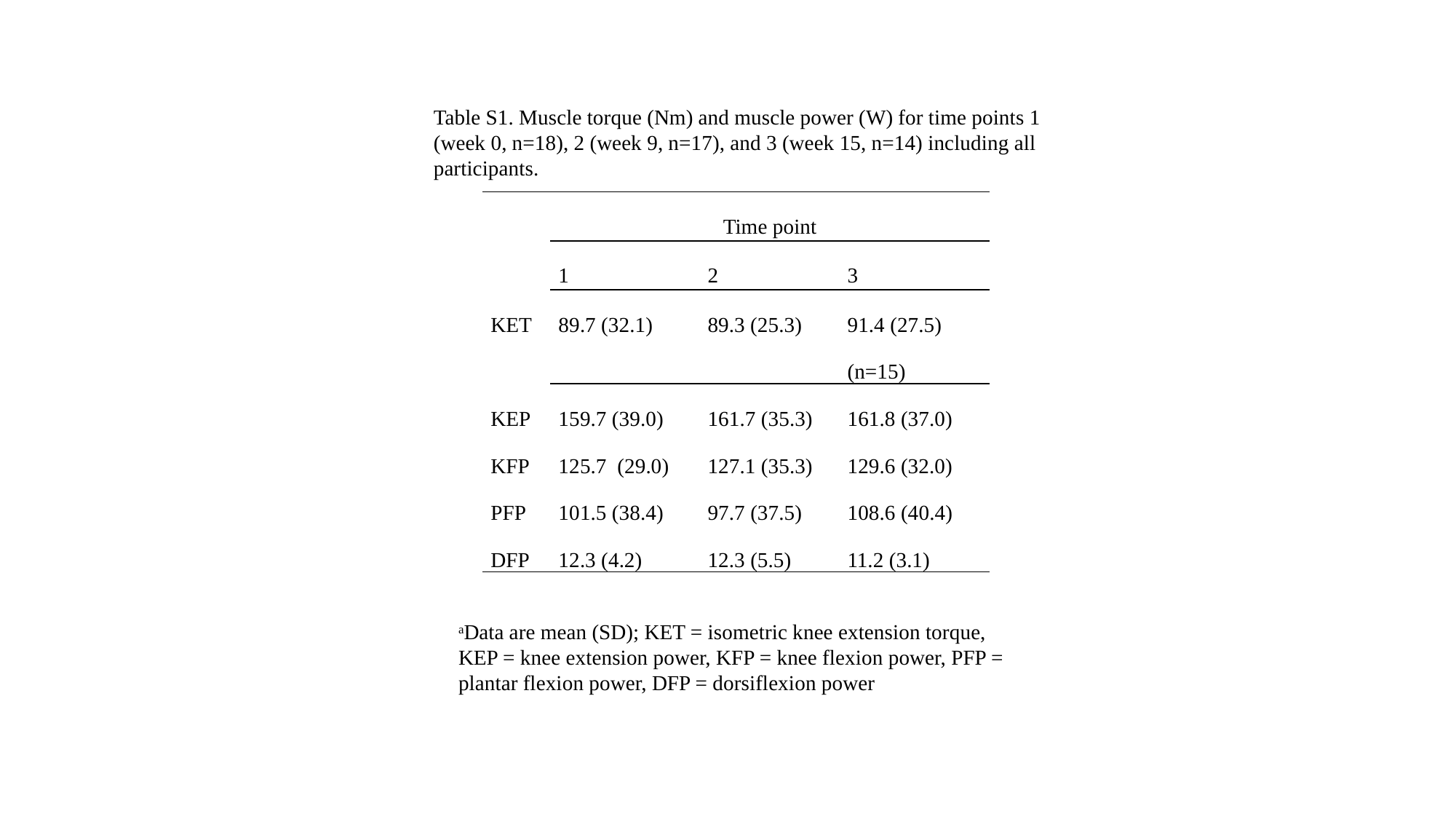

Table S1. Muscle torque (Nm) and muscle power (W) for time points 1 (week 0, n=18), 2 (week 9, n=17), and 3 (week 15, n=14) including all participants.
| | Time point | | |
| --- | --- | --- | --- |
| | 1 | 2 | 3 |
| KET | 89.7 (32.1) | 89.3 (25.3) | 91.4 (27.5) (n=15) |
| KEP | 159.7 (39.0) | 161.7 (35.3) | 161.8 (37.0) |
| KFP | 125.7 (29.0) | 127.1 (35.3) | 129.6 (32.0) |
| PFP | 101.5 (38.4) | 97.7 (37.5) | 108.6 (40.4) |
| DFP | 12.3 (4.2) | 12.3 (5.5) | 11.2 (3.1) |
aData are mean (SD); KET = isometric knee extension torque, KEP = knee extension power, KFP = knee flexion power, PFP = plantar flexion power, DFP = dorsiflexion power

## Slide 2
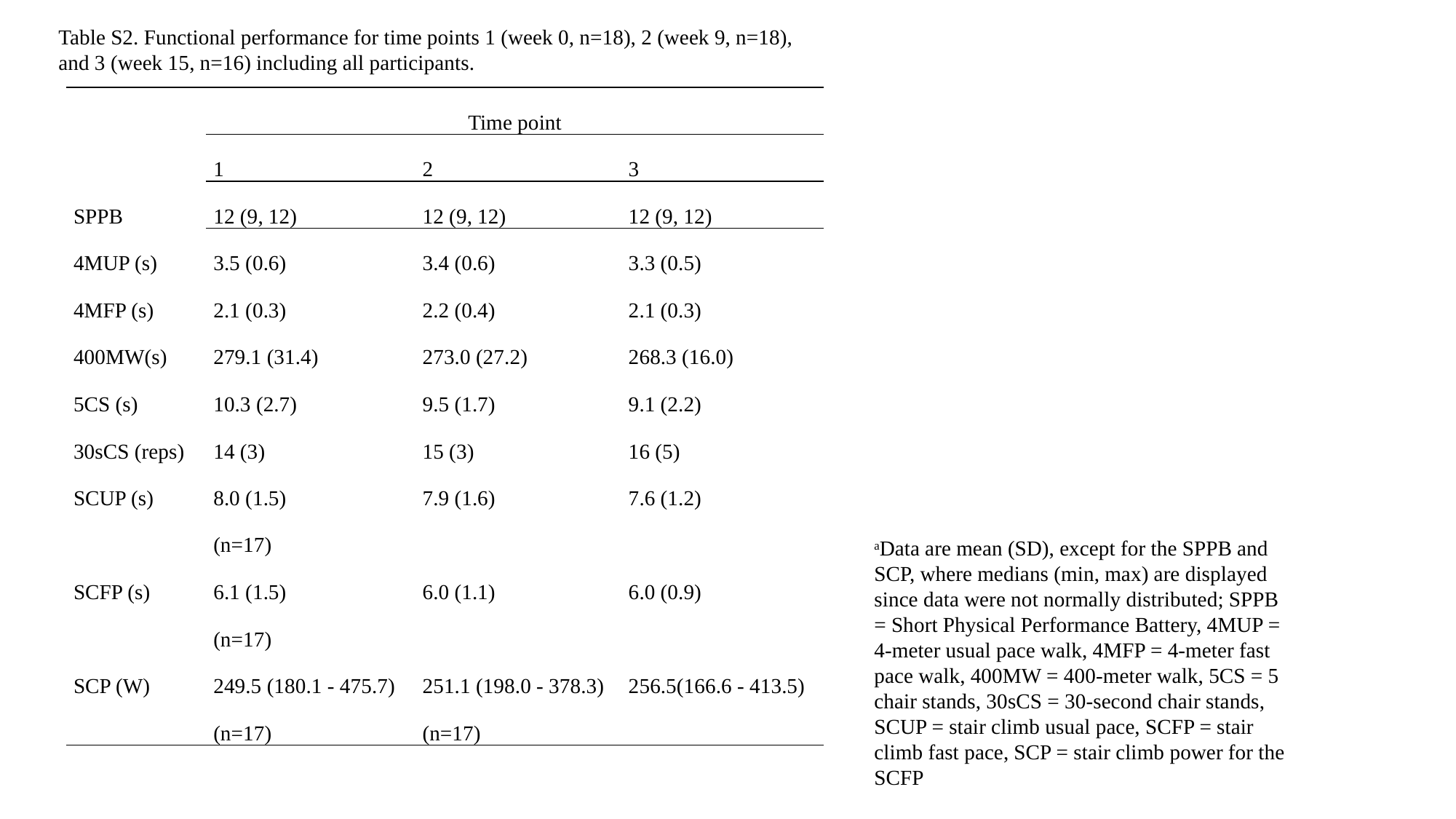

Table S2. Functional performance for time points 1 (week 0, n=18), 2 (week 9, n=18), and 3 (week 15, n=16) including all participants.
| | Time point | | |
| --- | --- | --- | --- |
| | 1 | 2 | 3 |
| SPPB | 12 (9, 12) | 12 (9, 12) | 12 (9, 12) |
| 4MUP (s) | 3.5 (0.6) | 3.4 (0.6) | 3.3 (0.5) |
| 4MFP (s) | 2.1 (0.3) | 2.2 (0.4) | 2.1 (0.3) |
| 400MW(s) | 279.1 (31.4) | 273.0 (27.2) | 268.3 (16.0) |
| 5CS (s) | 10.3 (2.7) | 9.5 (1.7) | 9.1 (2.2) |
| 30sCS (reps) | 14 (3) | 15 (3) | 16 (5) |
| SCUP (s) | 8.0 (1.5) (n=17) | 7.9 (1.6) | 7.6 (1.2) |
| SCFP (s) | 6.1 (1.5) (n=17) | 6.0 (1.1) | 6.0 (0.9) |
| SCP (W) | 249.5 (180.1 - 475.7) (n=17) | 251.1 (198.0 - 378.3) (n=17) | 256.5(166.6 - 413.5) |
aData are mean (SD), except for the SPPB and SCP, where medians (min, max) are displayed since data were not normally distributed; SPPB = Short Physical Performance Battery, 4MUP = 4-meter usual pace walk, 4MFP = 4-meter fast pace walk, 400MW = 400-meter walk, 5CS = 5 chair stands, 30sCS = 30-second chair stands, SCUP = stair climb usual pace, SCFP = stair climb fast pace, SCP = stair climb power for the SCFP

## Slide 3
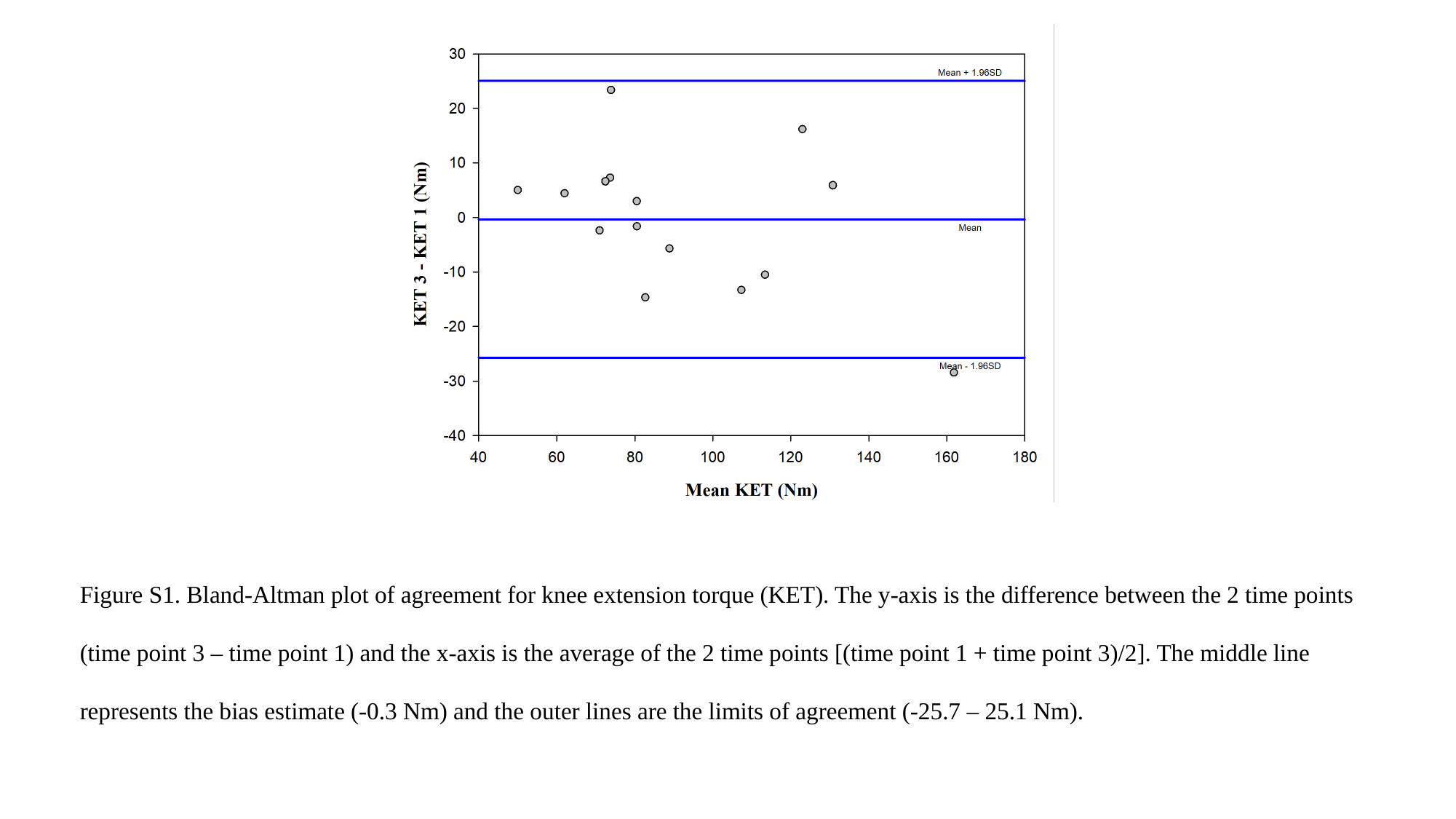

Figure S1. Bland-Altman plot of agreement for knee extension torque (KET). The y-axis is the difference between the 2 time points (time point 3 – time point 1) and the x-axis is the average of the 2 time points [(time point 1 + time point 3)/2]. The middle line represents the bias estimate (-0.3 Nm) and the outer lines are the limits of agreement (-25.7 – 25.1 Nm).

## Slide 4
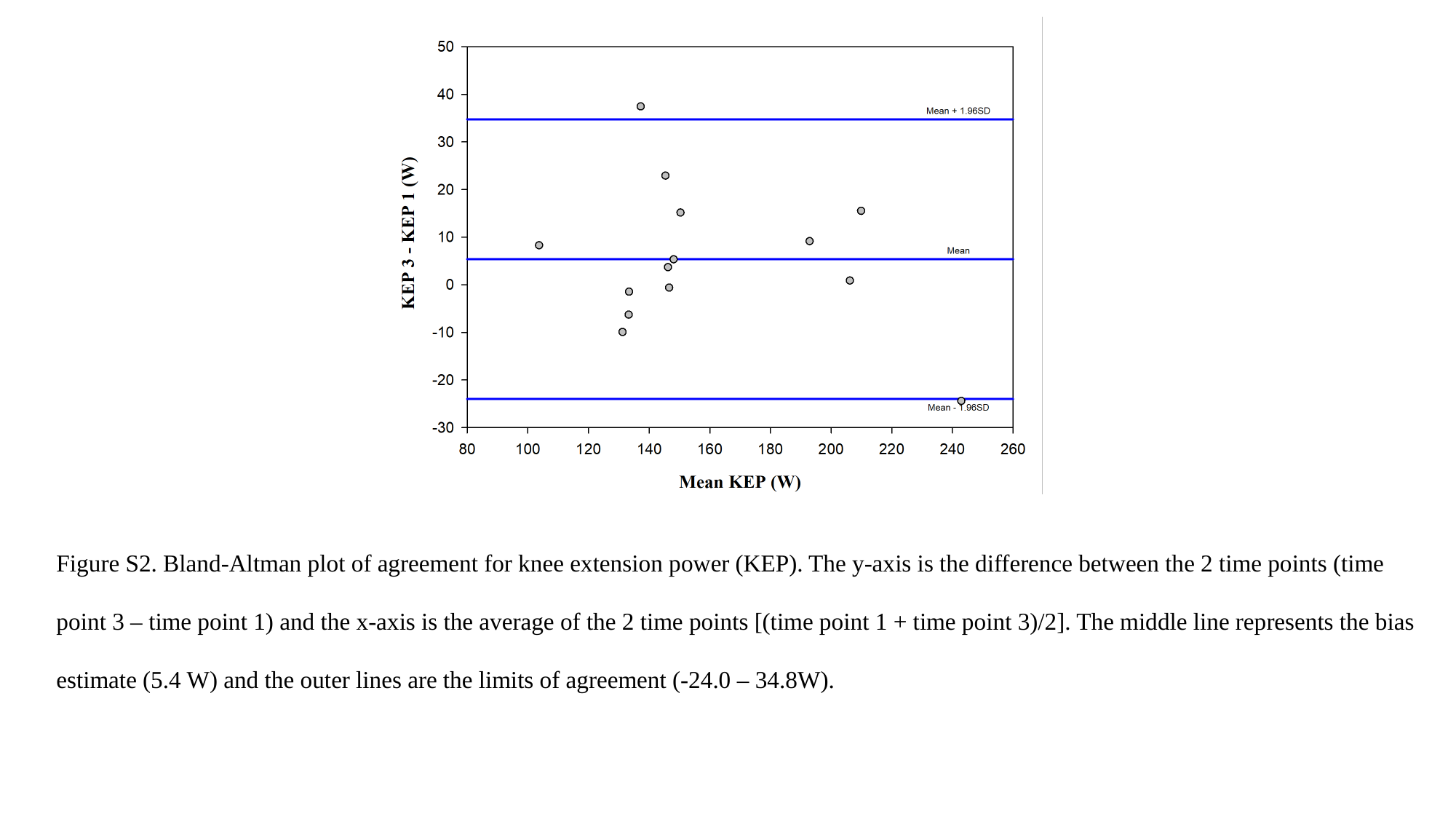

Figure S2. Bland-Altman plot of agreement for knee extension power (KEP). The y-axis is the difference between the 2 time points (time point 3 – time point 1) and the x-axis is the average of the 2 time points [(time point 1 + time point 3)/2]. The middle line represents the bias estimate (5.4 W) and the outer lines are the limits of agreement (-24.0 – 34.8W).

## Slide 5
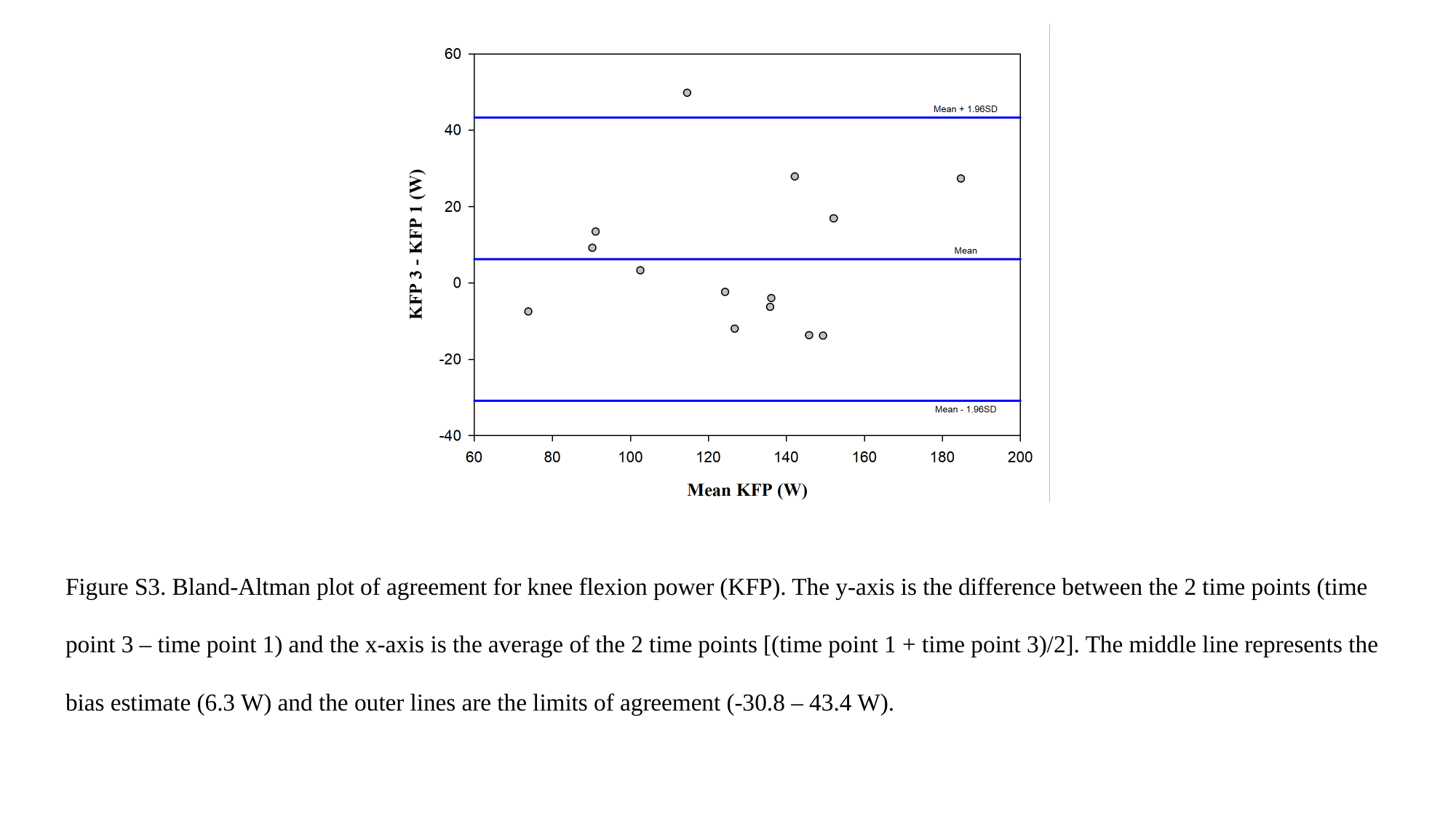

Figure S3. Bland-Altman plot of agreement for knee flexion power (KFP). The y-axis is the difference between the 2 time points (time point 3 – time point 1) and the x-axis is the average of the 2 time points [(time point 1 + time point 3)/2]. The middle line represents the bias estimate (6.3 W) and the outer lines are the limits of agreement (-30.8 – 43.4 W).

## Slide 6
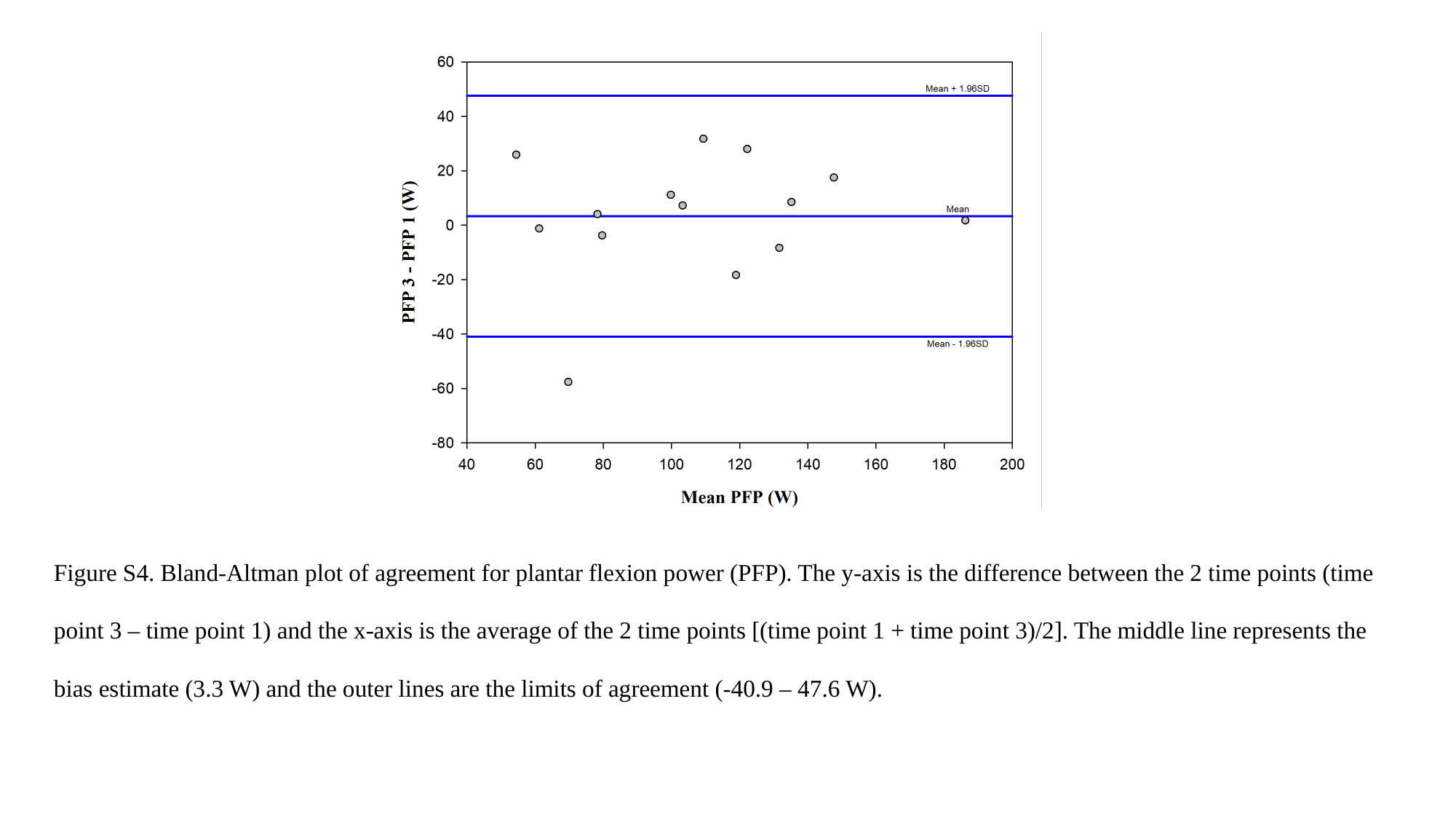

Figure S4. Bland-Altman plot of agreement for plantar flexion power (PFP). The y-axis is the difference between the 2 time points (time point 3 – time point 1) and the x-axis is the average of the 2 time points [(time point 1 + time point 3)/2]. The middle line represents the bias estimate (3.3 W) and the outer lines are the limits of agreement (-40.9 – 47.6 W).

## Slide 7
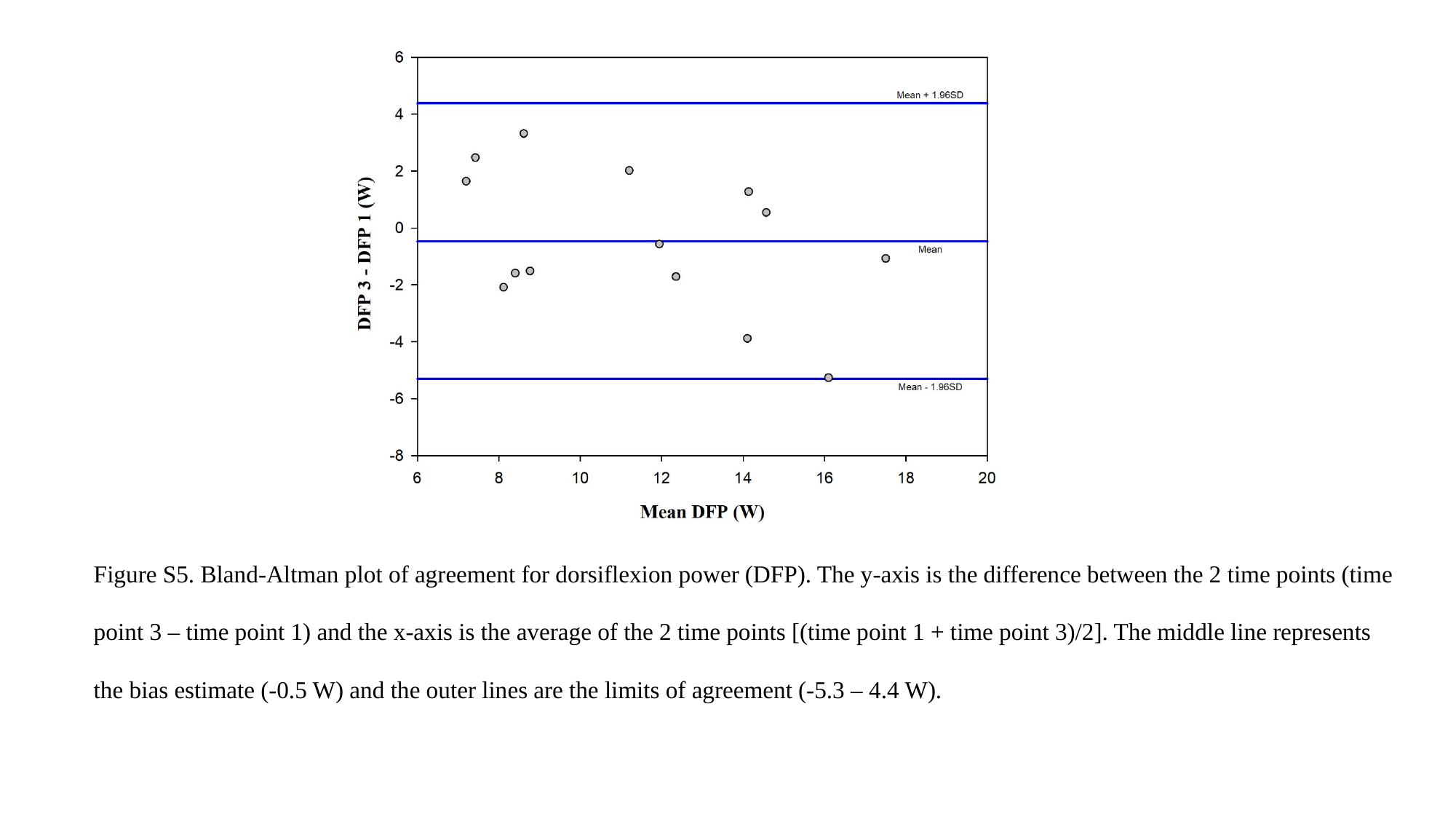

Figure S5. Bland-Altman plot of agreement for dorsiflexion power (DFP). The y-axis is the difference between the 2 time points (time point 3 – time point 1) and the x-axis is the average of the 2 time points [(time point 1 + time point 3)/2]. The middle line represents the bias estimate (-0.5 W) and the outer lines are the limits of agreement (-5.3 – 4.4 W).

## Slide 8
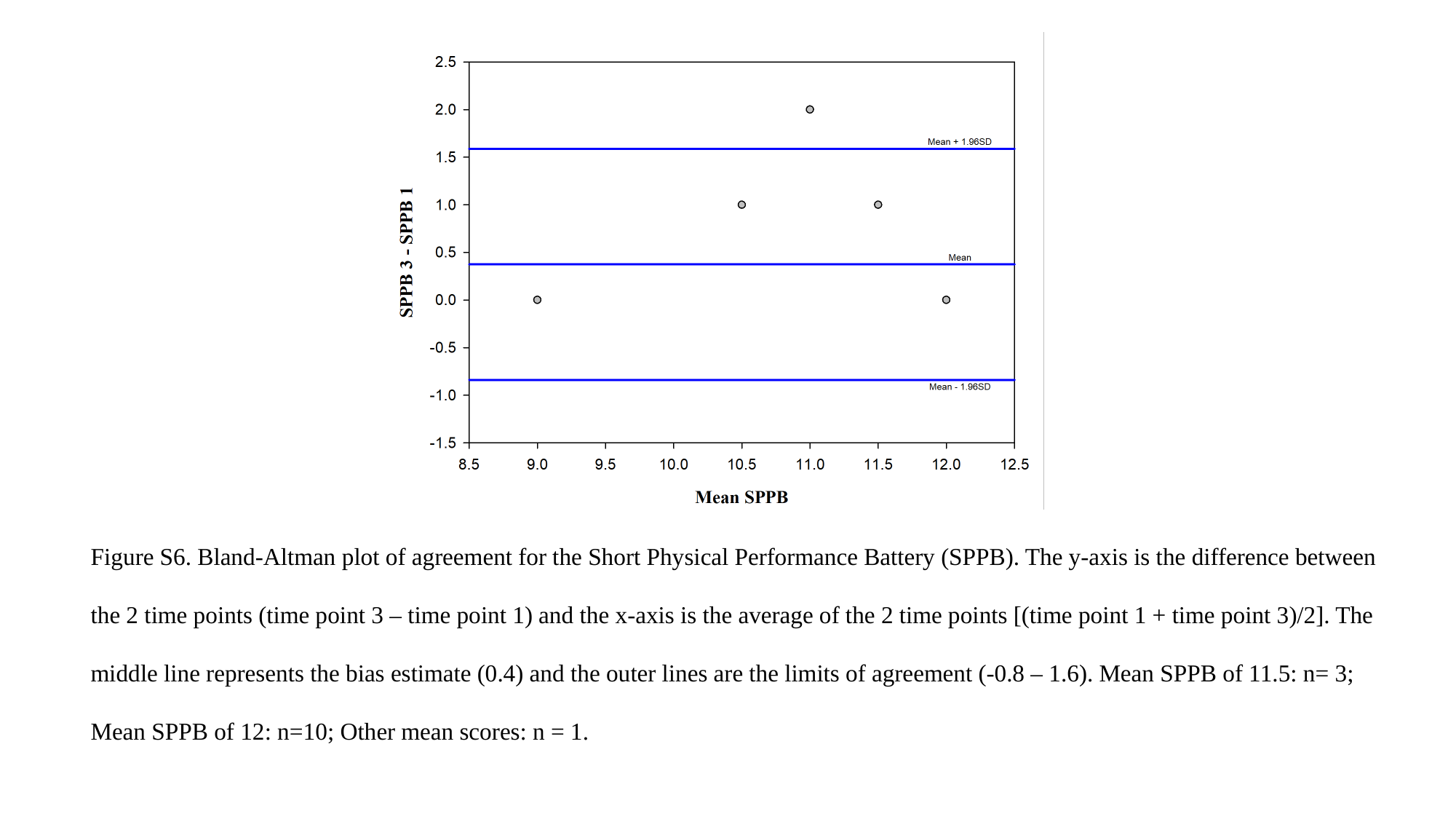

Figure S6. Bland-Altman plot of agreement for the Short Physical Performance Battery (SPPB). The y-axis is the difference between the 2 time points (time point 3 – time point 1) and the x-axis is the average of the 2 time points [(time point 1 + time point 3)/2]. The middle line represents the bias estimate (0.4) and the outer lines are the limits of agreement (-0.8 – 1.6). Mean SPPB of 11.5: n= 3; Mean SPPB of 12: n=10; Other mean scores: n = 1.

## Slide 9
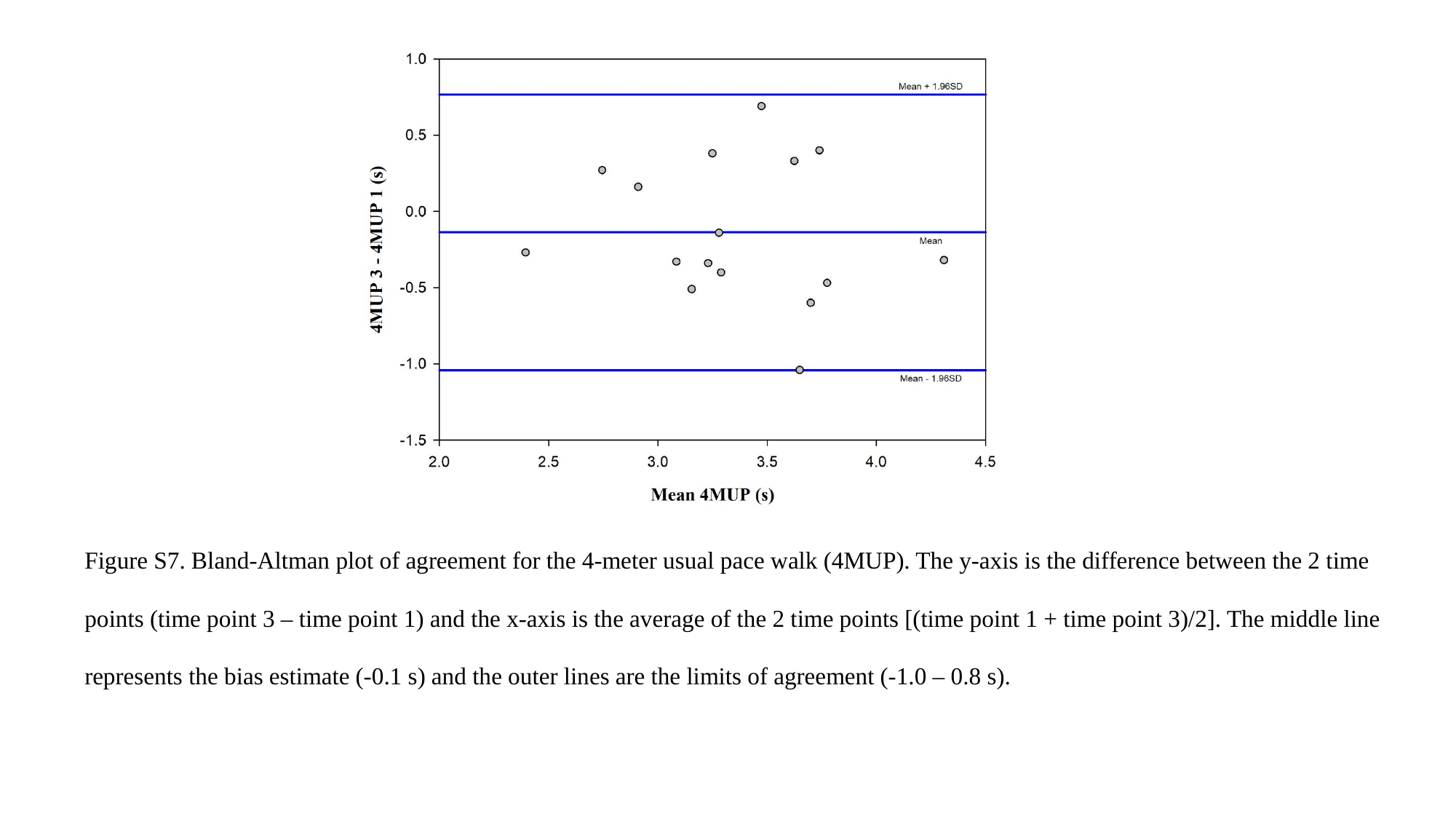

Figure S7. Bland-Altman plot of agreement for the 4-meter usual pace walk (4MUP). The y-axis is the difference between the 2 time points (time point 3 – time point 1) and the x-axis is the average of the 2 time points [(time point 1 + time point 3)/2]. The middle line represents the bias estimate (-0.1 s) and the outer lines are the limits of agreement (-1.0 – 0.8 s).

## Slide 10
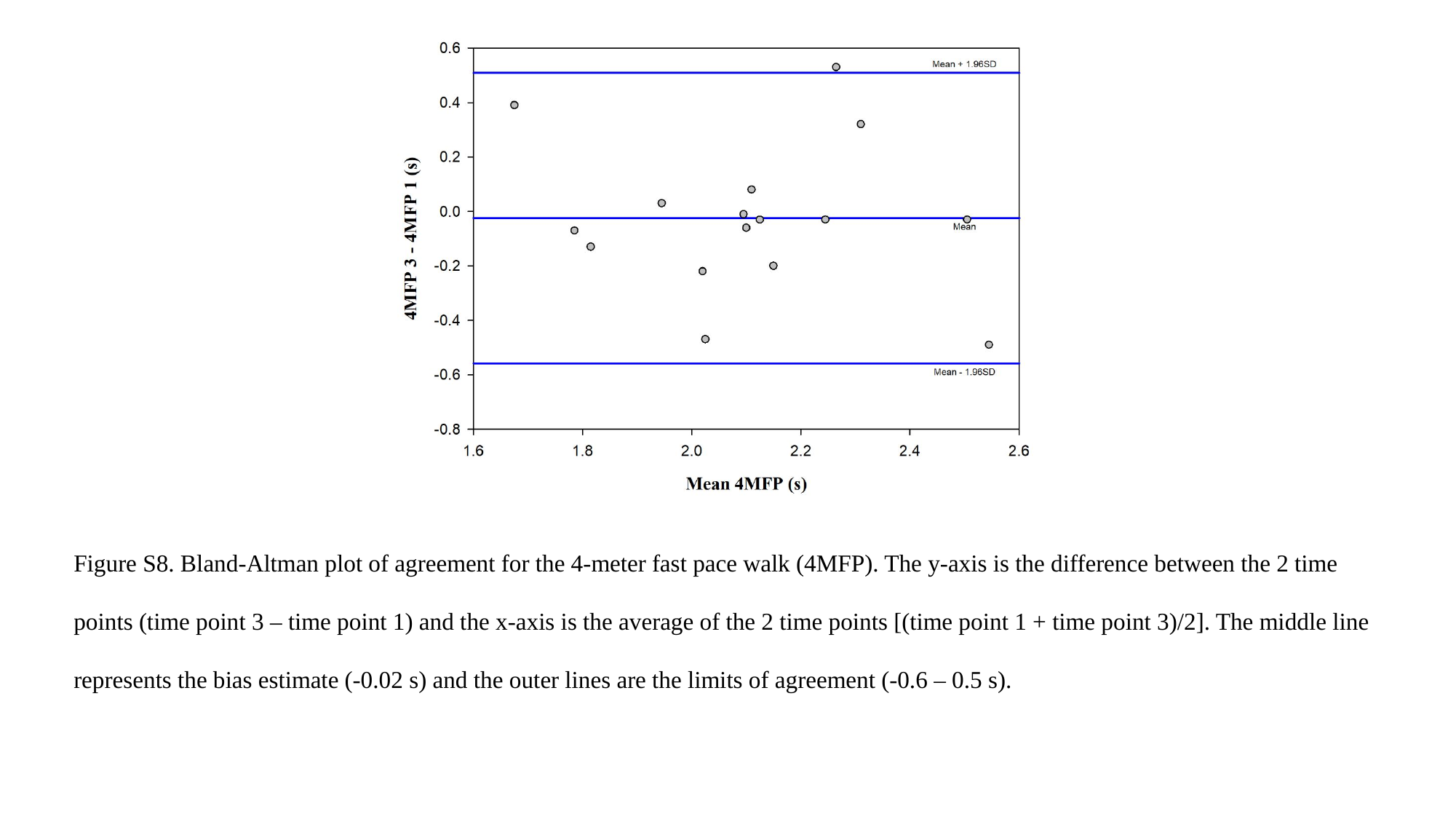

Figure S8. Bland-Altman plot of agreement for the 4-meter fast pace walk (4MFP). The y-axis is the difference between the 2 time points (time point 3 – time point 1) and the x-axis is the average of the 2 time points [(time point 1 + time point 3)/2]. The middle line represents the bias estimate (-0.02 s) and the outer lines are the limits of agreement (-0.6 – 0.5 s).

## Slide 11
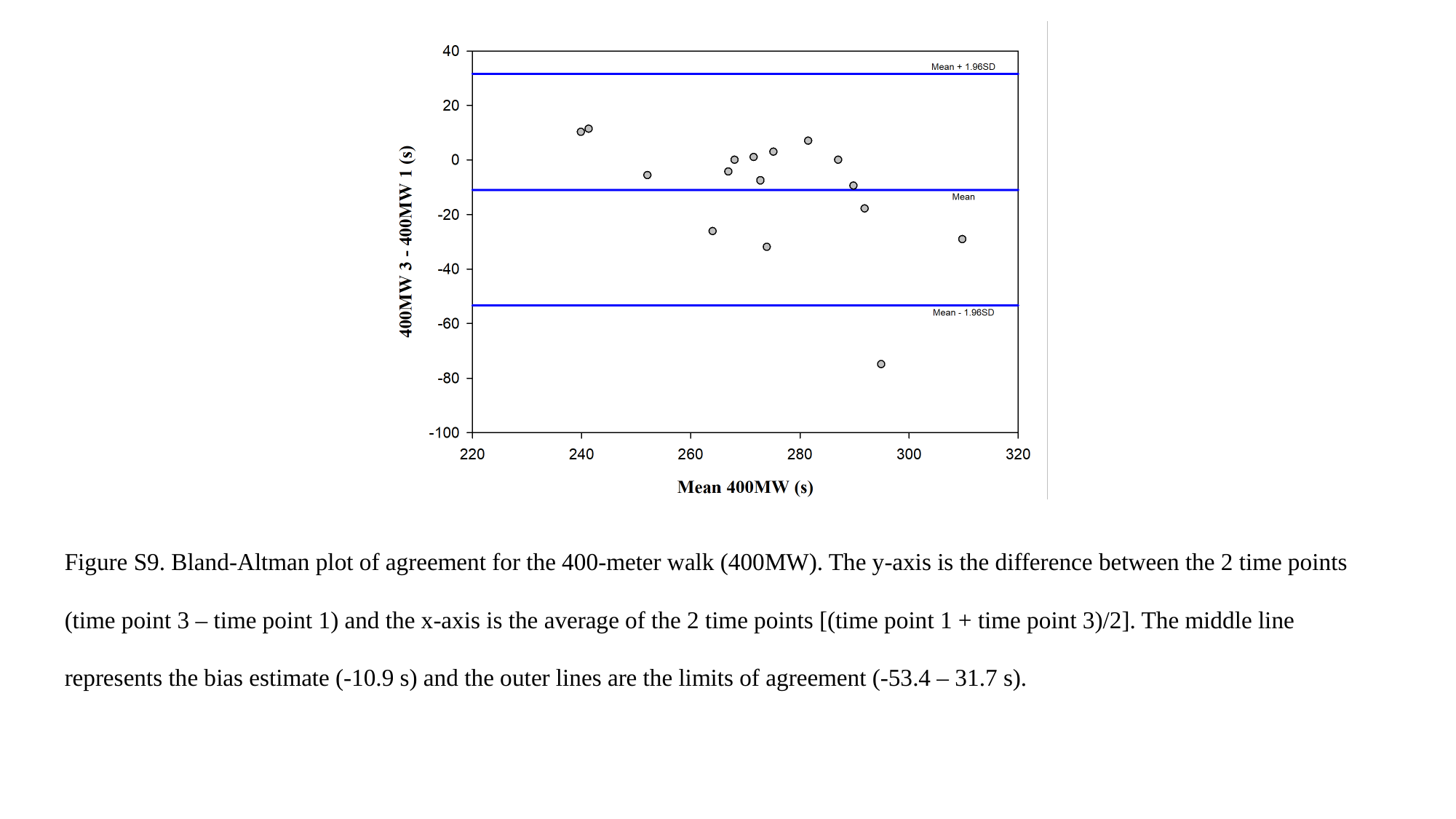

Figure S9. Bland-Altman plot of agreement for the 400-meter walk (400MW). The y-axis is the difference between the 2 time points (time point 3 – time point 1) and the x-axis is the average of the 2 time points [(time point 1 + time point 3)/2]. The middle line represents the bias estimate (-10.9 s) and the outer lines are the limits of agreement (-53.4 – 31.7 s).

## Slide 12
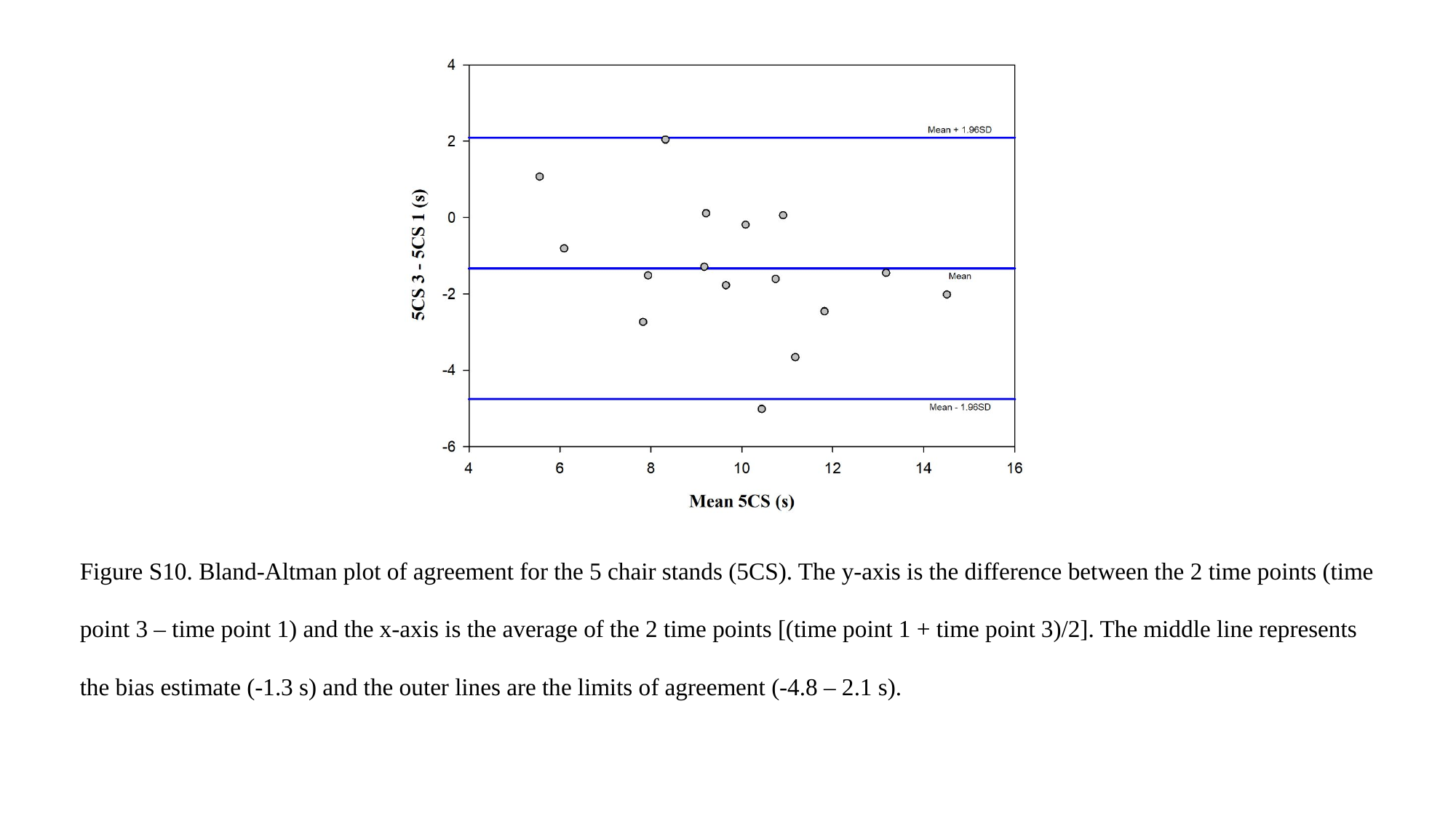

Figure S10. Bland-Altman plot of agreement for the 5 chair stands (5CS). The y-axis is the difference between the 2 time points (time point 3 – time point 1) and the x-axis is the average of the 2 time points [(time point 1 + time point 3)/2]. The middle line represents the bias estimate (-1.3 s) and the outer lines are the limits of agreement (-4.8 – 2.1 s).

## Slide 13
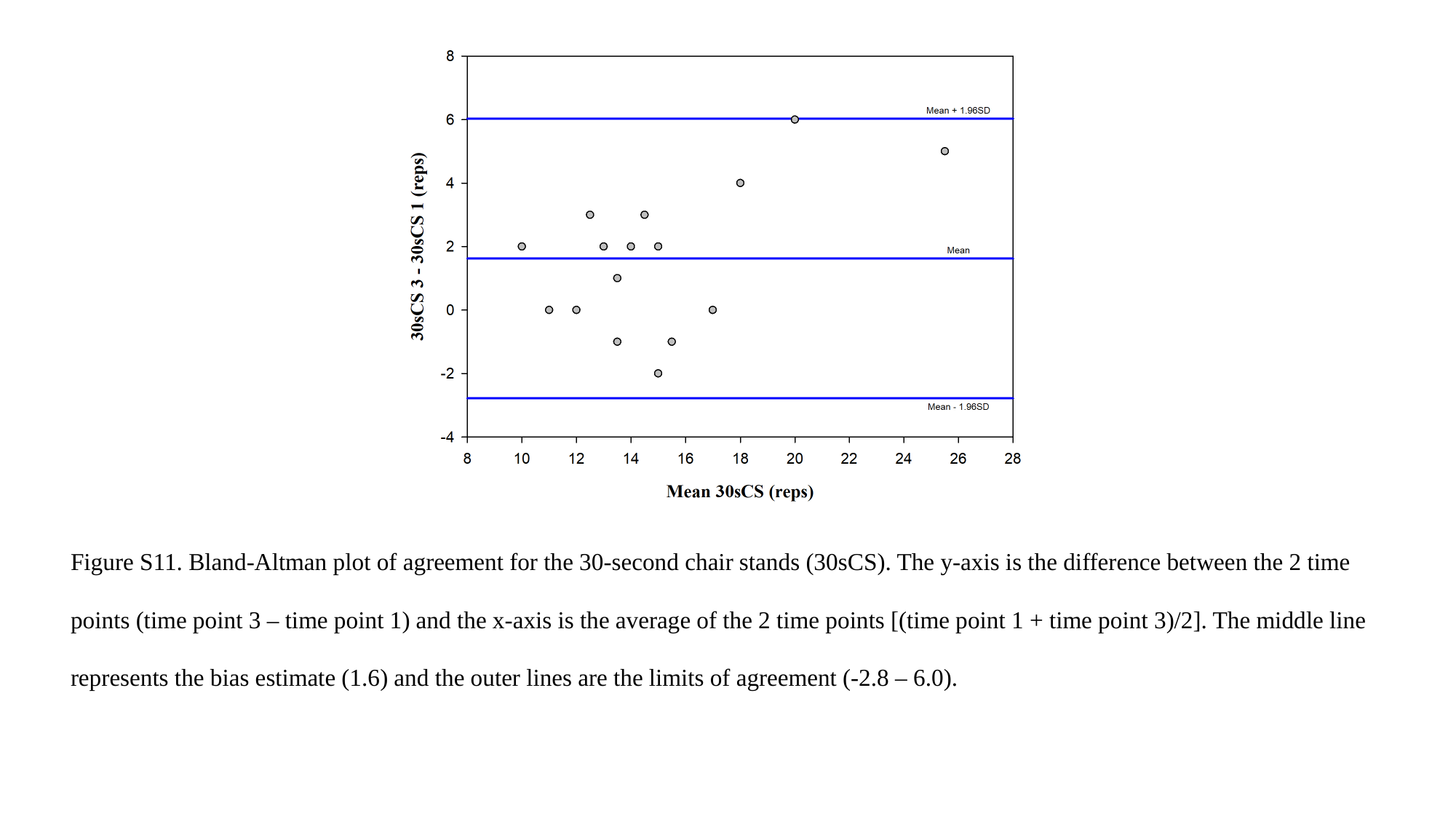

Figure S11. Bland-Altman plot of agreement for the 30-second chair stands (30sCS). The y-axis is the difference between the 2 time points (time point 3 – time point 1) and the x-axis is the average of the 2 time points [(time point 1 + time point 3)/2]. The middle line represents the bias estimate (1.6) and the outer lines are the limits of agreement (-2.8 – 6.0).

## Slide 14
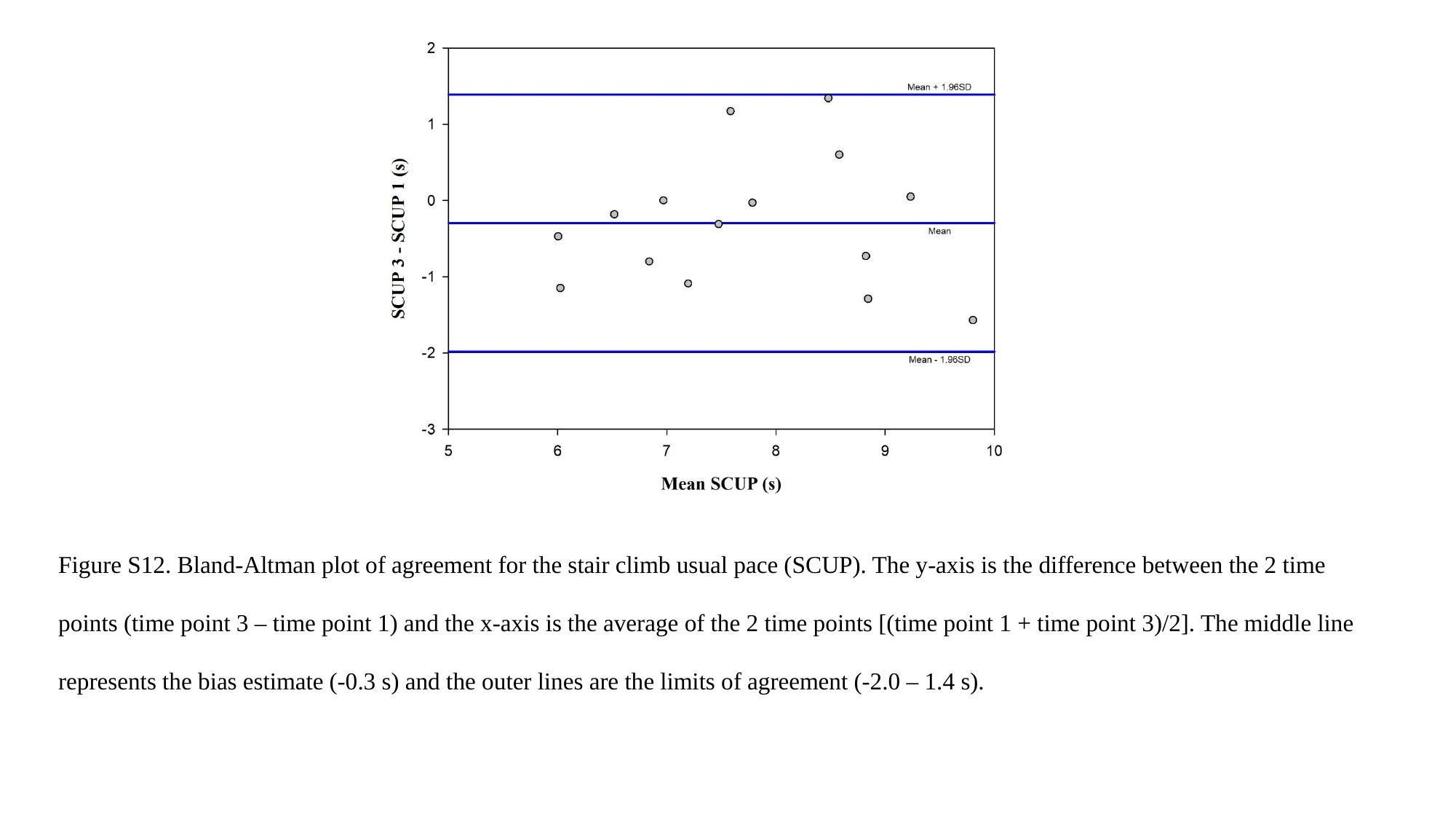

Figure S12. Bland-Altman plot of agreement for the stair climb usual pace (SCUP). The y-axis is the difference between the 2 time points (time point 3 – time point 1) and the x-axis is the average of the 2 time points [(time point 1 + time point 3)/2]. The middle line represents the bias estimate (-0.3 s) and the outer lines are the limits of agreement (-2.0 – 1.4 s).

## Slide 15
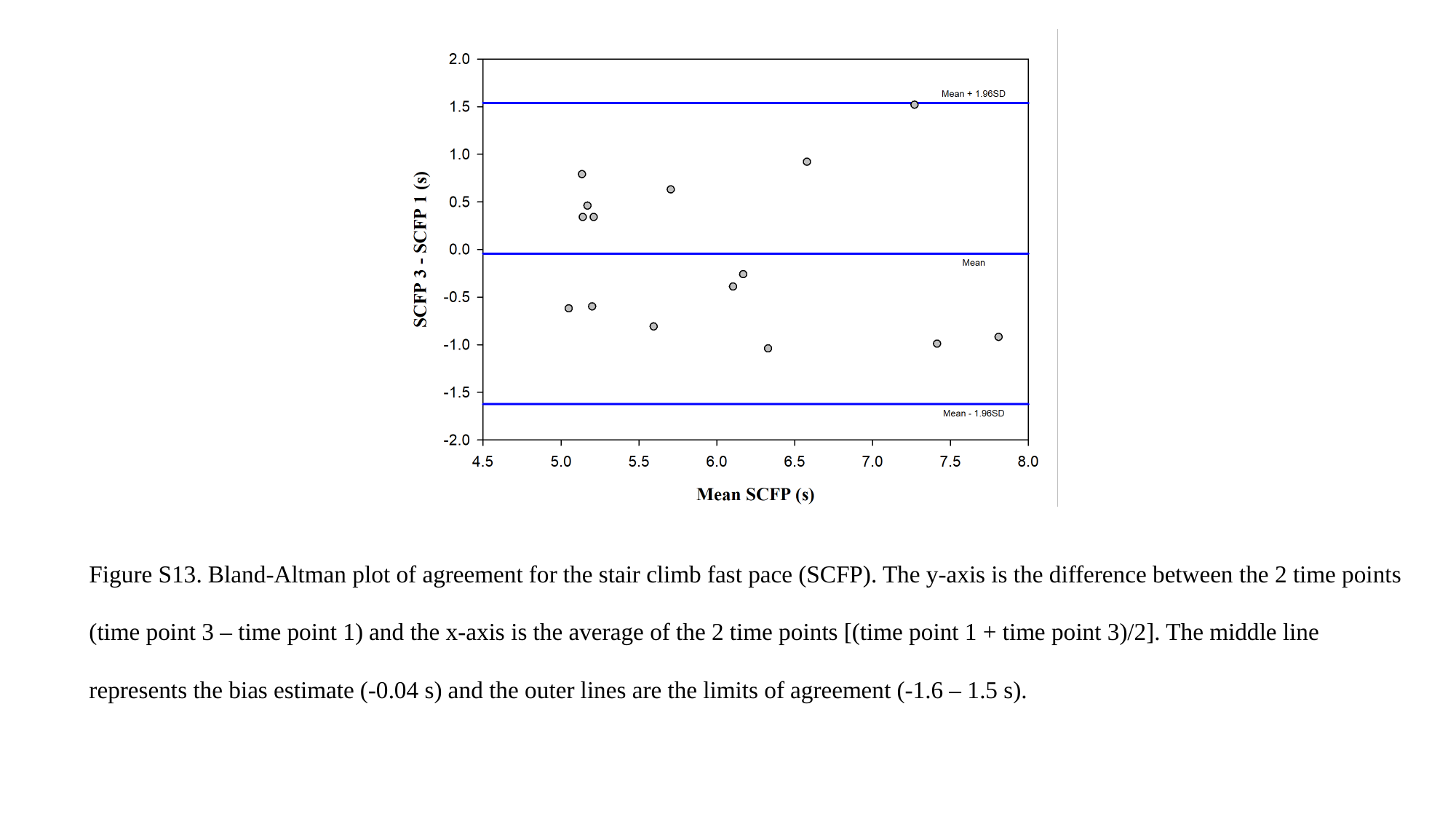

Figure S13. Bland-Altman plot of agreement for the stair climb fast pace (SCFP). The y-axis is the difference between the 2 time points (time point 3 – time point 1) and the x-axis is the average of the 2 time points [(time point 1 + time point 3)/2]. The middle line represents the bias estimate (-0.04 s) and the outer lines are the limits of agreement (-1.6 – 1.5 s).

## Slide 16
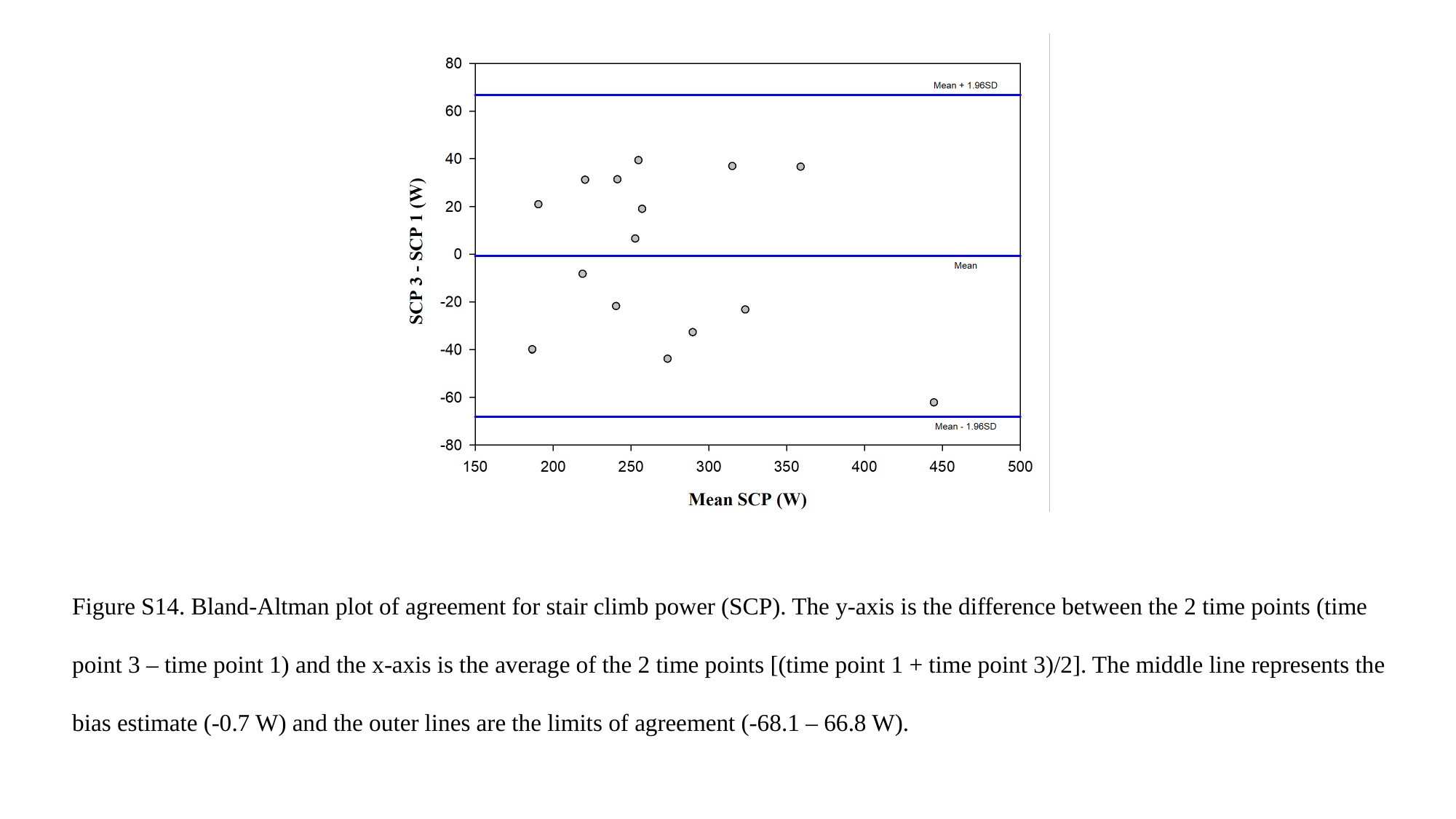

Figure S14. Bland-Altman plot of agreement for stair climb power (SCP). The y-axis is the difference between the 2 time points (time point 3 – time point 1) and the x-axis is the average of the 2 time points [(time point 1 + time point 3)/2]. The middle line represents the bias estimate (-0.7 W) and the outer lines are the limits of agreement (-68.1 – 66.8 W).
